# Effect of pannus formation on the prosthetic heart valve: In vitro demonstration using particle image velocimetry

**DOI:** 10.1371/journal.pone.0199792

**Published:** 2018-06-28

**Authors:** Hojin Ha, Hyun Jung Koo, Hyung Kyu Huh, Guk Bae Kim, Jihoon Kweon, Namkug Kim, Young-Hak Kim, Joon-Won Kang, Tae-Hwan Lim, Jae-Kwan Song, Sang Joon Lee, Dong Hyun Yang

**Affiliations:** 1 Department of Mechanical and Biomedical Engineering, Kangwon National University, Chuncheon, South Korea; 2 Department of Radiology, University of Ulsan College of Medicine, Asan Medical Center, Seoul, South Korea; 3 Department of Mechanical Engineering, Pohang University of Science and Technology, Pohang, South Korea; 4 Asan Institute of Life Science, Asan Medical Center, University of Ulsan College of Medicine, Seoul, South Korea; 5 Department of Cardiology, University of Ulsan College of Medicine, Asan Medical Center, Seoul, South Korea; 6 Department of Convergence Medicine, University of Ulsan College of Medicine, Asan Medical Center, Seoul, South Korea; Cincinnati Children's Hospital Medical Center, UNITED STATES

## Abstract

Although hemodynamic influence of the subprosthetic tissue, termed as pannus, may contribute to prosthetic aortic valve dysfunction, the relationship between pannus extent and hemodynamics in the prosthetic valve has rarely been reported. We investigated the fluid dynamics of pannus formation using in vitro experiments with particle image velocimetry. Subvalvular pannus formation caused substantial changes in prosthetic valve transvalvular peak velocity, transvalvular pressure gradient (TPG) and opening angle. Maximum flow velocity and corresponding TPG were mostly affected by pannus width. When the pannus width was 25% of the valve diameter, pannus formation elevated TPG to >2.5 times higher than that without pannus formation. Opening dysfunction was observed only for a pannus involvement angle of 360°. Although circumferential pannus with an involvement angle of 360° decreased the opening angle of the valve from approximately 82° to 58°, eccentric pannus with an involvement angle of 180° did not induce valve opening dysfunction. The pannus involvement angle largely influenced the velocity flow field at the aortic sinus and corresponding hemodynamic indices, including wall shear stress, principal shear stress and viscous energy loss distributions. Substantial discrepancy between the velocity-based TPG estimation and direct pressure measurements was observed for prosthetic valve flow with pannus formation.

## Introduction

**Fig 1:**
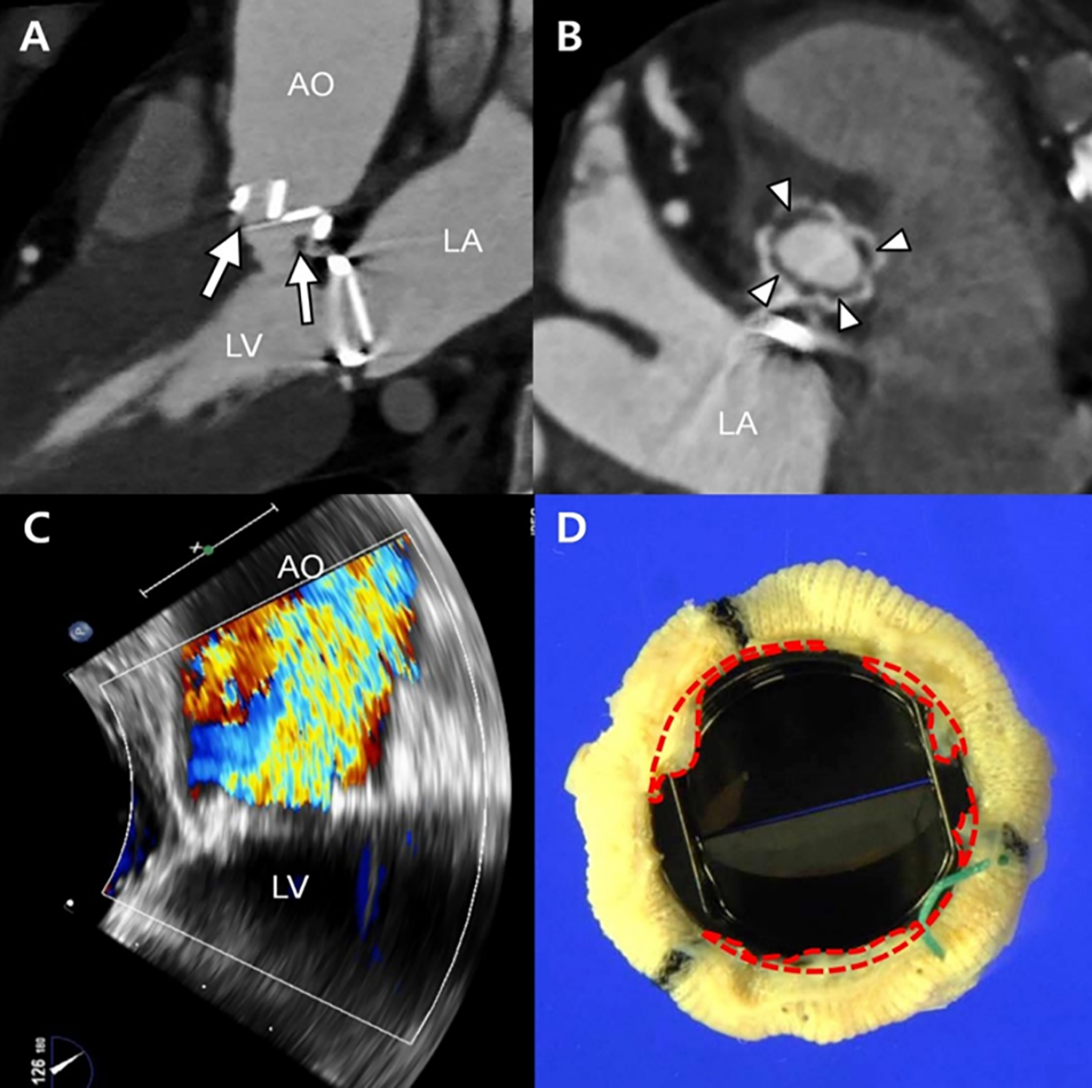
Representative images showing a subvalvular pannus involving the prosthetic aortic valve. (A) Multiplanar CT reformation in profile view showing the subvalvular soft tissue below the posterior prosthetic valve (arrow) and decreased opening angle of the posterior leaflet. (B) Aortic valve in-plane view on CT showing circumferential involvement of the soft-tissue lesions (arrowheads) below the prosthetic valve around the strut. (C) High peak pressure gradient (140/100 mmHg) and peak velocity (6.2 m/s) through the aortic valve are noted on echocardiography. (D) The surgical specimen of the prosthetic valve showing the circumferential fibrous soft tissue, named as pannus (areas in the red dashed line), in the subvalvular area. The lesion is corresponded with the lucent area (B, arrows) detected on CT. AO, ascending aorta; LA, left atrium; LV, left ventricle.

Growth of abnormal tissue around a prosthetic heart valve, termed as pannus formation, is one of the important causes for prosthetic valve dysfunction that may require repeated open heart surgery [1–4]. Protrusion of the pannus into the valve orifice area may disturb blood flow through the valve and finally result in prosthetic valve stenosis. Conventional imaging methodologies, such as fluoroscopy or echocardiography, are limited to demonstrating the pannus itself. Alternatively, the opening angle of the prosthetic valve or flow velocity through the prosthetic valve were measured with fluoroscopy or echocardiography for diagnosing pannus formation indirectly. Recently, advancement in computed tomography (CT) facilitated more precise visualization and quantification of the shape and extent of the pannus (Fig 1) [2, 4].

Despite CT demonstration of pannus formation, the relationship between prosthetic valve dysfunction and the extent of pannus involvement remains unclear. Difficulties remain in making a clinical decision in patients with CT-identified pannus. Most of all, the relationship between the extent of pannus and elevation of the pressure gradient through the prosthetic valve, indicating obstruction of valvular blood flow, has not been clarified thus far. Therefore, diagnosis and decision-making for surgical intervention suffer from insufficient knowledge of pannus formation in patients with varying size and motions of the prosthetic valve and left ventricle.

Recently, because of advancement in 3D printing and flow analysis technology, in vitro experiments with an artificial flow phantom have been used as an effective method for simulating fluid mechanical aspects of blood flow [5–7]. For example, a combination of the artificial flow phantom and velocity field measurement, such as particle image velocimetry (PIV), has been widely used to reveal hemodynamic characteristics in a blood vessel with stenosis, aneurysm, or a stent [8–12]. In particular, hemodynamic characteristics of prosthetic valve flow have also been investigated using an in vitro flow phantom with the PIV technique [13–15].

In this study, we aimed to investigate the effect of pannus formation on prosthetic valve mechanical function and its hemodynamic characteristics. In vitro flow phantom demonstration with PIV measurement was used for investigating the effect of pannus formation on the prosthetic valve and blood flow under controlled conditions. While the in-vitro experiments with the simplified aortic geometry may not exactly reproduce the in vivo conditions, but they would provide a first approximation of the effect of pannus formation on the hemodynamic characteristics.

## Materials and methods

### Representative patient with pannus formation

Regarding to the representative patient shown in Fig 1, all methods were carried out in accordance with relevant guidelines and regulations. The institutional review board of Asan Medical Center (Seoul, South Korea) approved retrospective investigation of the patient with pannus formation. All clinical investigation have been conducted according to the principles expressed in the Declaration of Helsinki. The need for informed consent from the patients was waived for the retrospective study. We searched the echocardiography and CT databases to identify patients with pannus formation in the prosthetic aortic valve. The electronic medical records, echocardiographic findings, and CT images were thoroughly reviewed. Pannus formation was suspected based on the following clinical findings: (a) abnormally increased peak velocity or pressure gradient on follow-up echocardiography and (b) subvalvular soft tissue detected on cardiac CT as a result of any cause [16]. After reviewing all reconstructed CT data and volume-rendered images, representative patient who exhibited a definite soft-tissue lesion on CT was identified.

Echocardiography images of the representative patient were obtained by experienced cardiologists, and the images were recorded as video files. Using an ultrasound system with a 3–5 MHz, real-time, echocardiographic transducer (Sonos 7500; Philips Medical Systems, Andover, MA; Vivid 7, General Electric, Waukesha, WI), conventional two-dimensional and Doppler echocardiography images were obtained. After a routine echocardiographic examination, additional Doppler B- and M-modes or 3D imaging were performed, if clinically needed [17, 18]. The echocardiographic left ventricular functional parameters were then evaluated.

Cardiac CT of the representative patient was performed using a second-generation dual-source CT scanner (Somatom Definition Flash; Siemens Medical Solutions, Forchheim, Germany). If there was no contraindication, 2.5 mg bisoprolol (Concor; Merck, Darmstadt, Germany) was orally administered to patients with heart rates >75 beats per minute, 1 h prior to the CT examination. Using a power injector (Stellant D; Medrad, Indianola, PA), a bolus of 60–80 ml of nonionic, iodinated contrast material (Iomeron; Bracco Imaging SpA, Milan, Italy) was injected at a rate of 4.0 ml/s, followed by 40 ml of a 30:70 mixture of contrast and saline. The bolus tracking method (ascending aorta; trigger threshold level, 100 HU; scan delay, 8 s) was used to obtain CT images. Retrospective electrocardiogram-gated scanning was applied with tube current modulation (dose pulsing windows, 30%–80% of the R–R interval). The tube voltage and tube current–time product were adjusted based on the patient’s body size. The scan parameters were as follows: tube voltage, 80–120 kV; tube current, 240–450 mAs; pitch 0.17–0.38; detector collimation, 64 × 0.6 mm; and gantry rotation time, 280 ms. The mean dose–length product for cardiac CT was 1174.9 ± 436.7 mGy∙cm, and the effective dose was 16.2 ± 6.0 mSv.

### Fabrication of an aortic sinus model with a prosthetic valve

In this study, one representative geometry with various pannus formations was designed so that we can study how pannus formation changes the fluid dynamics in the prosthetic valves by simplifying other geometric and patient parameters. In vitro flow phantoms of an aortic model with a prosthetic valve were fabricated by referring to previous studies [15, 19], and minor changes were made to fit the model with the prosthetic valve we used in the study. Based on a representative patient who had pannus formation near their prosthetic valves and had partially obstructed the flow path from the left ventricle to the aorta due to the septum of the left ventricle, the aortic model with the partially obstructed inlet flow was also included (Fig 1).

**Fig 2:**
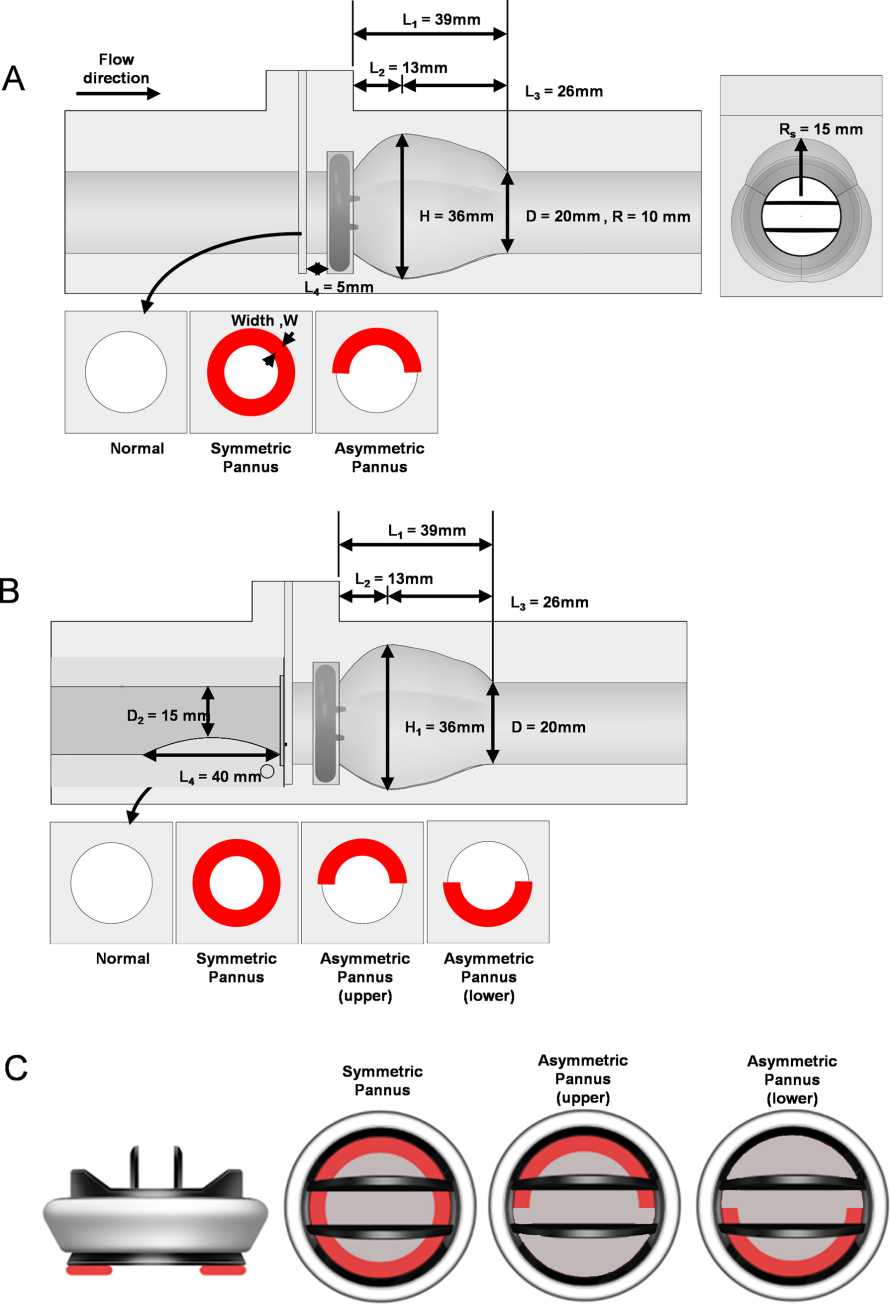
Geometry of the aortic sinus model with the prosthetic valve. Effects of pannus involvement (Pannus 360° vs. Pannus 180°) were compared to the normal condition without pannus formation. (A) The aortic sinus model with the straight inlet. (B) The aortic sinus model with 25% inlet obstruction mimicking partial flow path obstruction by the septum were constructed. (C) Illustration of the prosthetic valve with pannus formation (red shade).

An acrylic-based in vitro flow phantom was fabricated using a numerically controlled five-axis milling machine and then it was slightly polished. The aortic sinus model had a circular inlet and outlet 20 mm in diameter (Fig 2). The St. Jude prosthetic valve (19.6 mm inner diameter; St. Jude Medical, Inc., St. Paul, MN, USA) was installed in front of the aortic sinus. The pannus portion was installed 5 mm in front of the prosthetic valve.

In our study, 27 different cases were experimentally investigated by a combination of two different inlet conditions (a straight circular inlet and a 25% inlet obstruction mimicking partial obstruction of the flow path by the septum; Fig 2), two different pannus involvement angles (180° and 360°) and five different pannus widths. Based on the previous in-vivo studies [20], the extent of the pannus width was chosen from 0 to 25% of the diameter. Detailed experimental cases are summarized in Table 1.

**Table 1:** Demographics of experimental cases.

Case	Inlet	Pannus type	Involved angle (°)	Pannus width (mm)	W/D
SS0	Straight	Symmetric	360	0	0
SS1				1	0.05
SS2				2	0.10
SS3				3	0.15
SS4				4	0.20
SS5				5	0.25
SA1		Asymmetric	180	1	0.05
SA2				2	0.10
SA3				3	0.15
SA4				4	0.20
SA5				5	0.25
OS0	25% Obstruction	Symmetric	360	0	0
OS1				1	0.05
OS2				2	0.10
OS3				3	0.15
OS4				4	0.20
OS5				5	0.25
OAU1		Asymmetric (upper pannus)	180	1	0.05
OAU2				2	0.10
OAU3				3	0.15
OAU4				4	0.20
OAU5				5	0.25
OAD1		Asymmetric (lower pannus)		1	0.05
OAD2				2	0.10
OAD3				3	0.15
OAD4				4	0.20
OAD5				5	0.25

W, pannus width; D, diameter of the inlet vessel; SS, straight symmetric pannus; SA, straight asymmetric pannus; OS, obstructed inlet with symmetric pannus; OAU, obstructed inlet with asymmetric pannus at the upper wall; OAD, obstructed inlet with asymmetric pannus at the lower wall.

### Flow circuit system and pressure measurement

**Fig 3:**
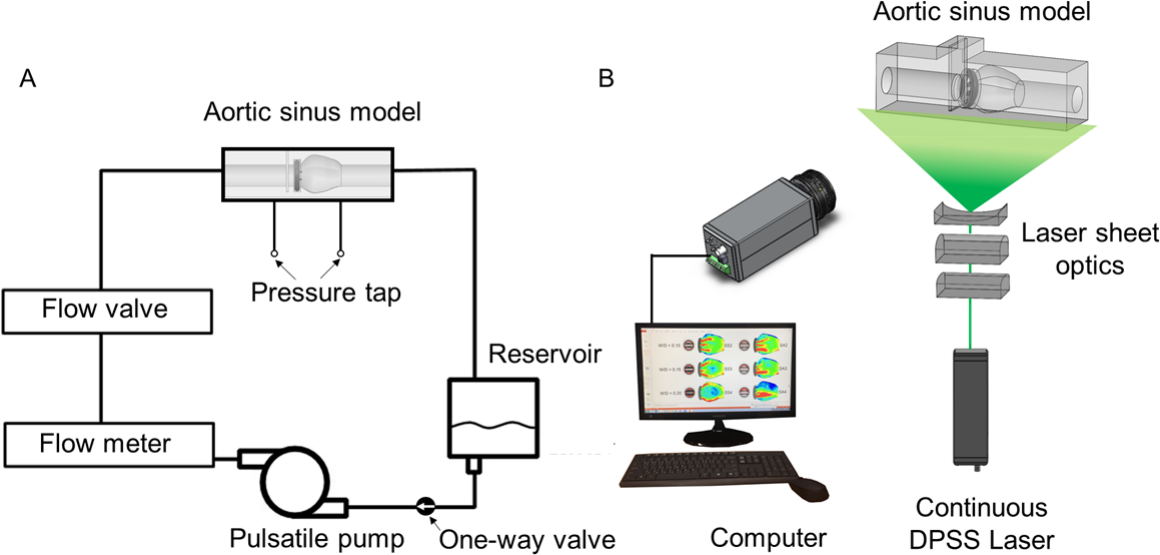
Schematics of the experimental set up. (A) Flow circuit system for PIV measurements. (B) Experimental set up for PIV measurement.

Fig 3A demonstrates the flow circulating system used in this study. A total of 9 L working fluid was prepared in an acrylic reservoir. A twin-pulsatile life support system (T-PLS®; New Heart Bio.BHK, Seoul, Korea) was used to circulate the working fluid at a constant pulsating frequency of 60 beats per minute. A one-way valve was installed at the inlet of the pulsatile pump to obtain a stable stroke volume by inhibiting possible regurgitant flow. The blood-analogue working fluid was composed of 79% saturated aqueous sodium iodide, 20% pure glycerol and 1% water (by volume). In our study, the kinematic viscosity of the working fluid was experimentally measured to be 2.8 × 10^−6^ m^2^/s using a rotational viscometer (DV-II+Pro, Brookfield Engineering Laboratories, Inc., Middleborough, MA, USA), which is within the range of human blood viscosity (2.8–3.8 × 10^−6^ m^2^/s) [21]. Using an Abbe refractometer (Atago, Tokyo, Japan), the refractive index was found to be 1.491 ± 0.001 at 25°C. It was in accordance with the experimental acrylic model and provided proper optical access without distortion. For PIV measurement, the working fluid was seeded with polymethyl methacrylate fluorescence particles (PMMA-Rhodamine B-particles; Dantec Dynamics, Skovlunde, Denmark), with a diameter of 20–50 μm, at approximately 0.01 wt% concentration. The seeding density of the particles was approximately 0.01% by weight. The effective size of the particle on the image was approximately 5 pixels, thereby resulting in approximately five particles per interrogation window (16 × 16 pixels). All experiments were performed at a controlled room temperature of 25°C.

**Fig 4:**
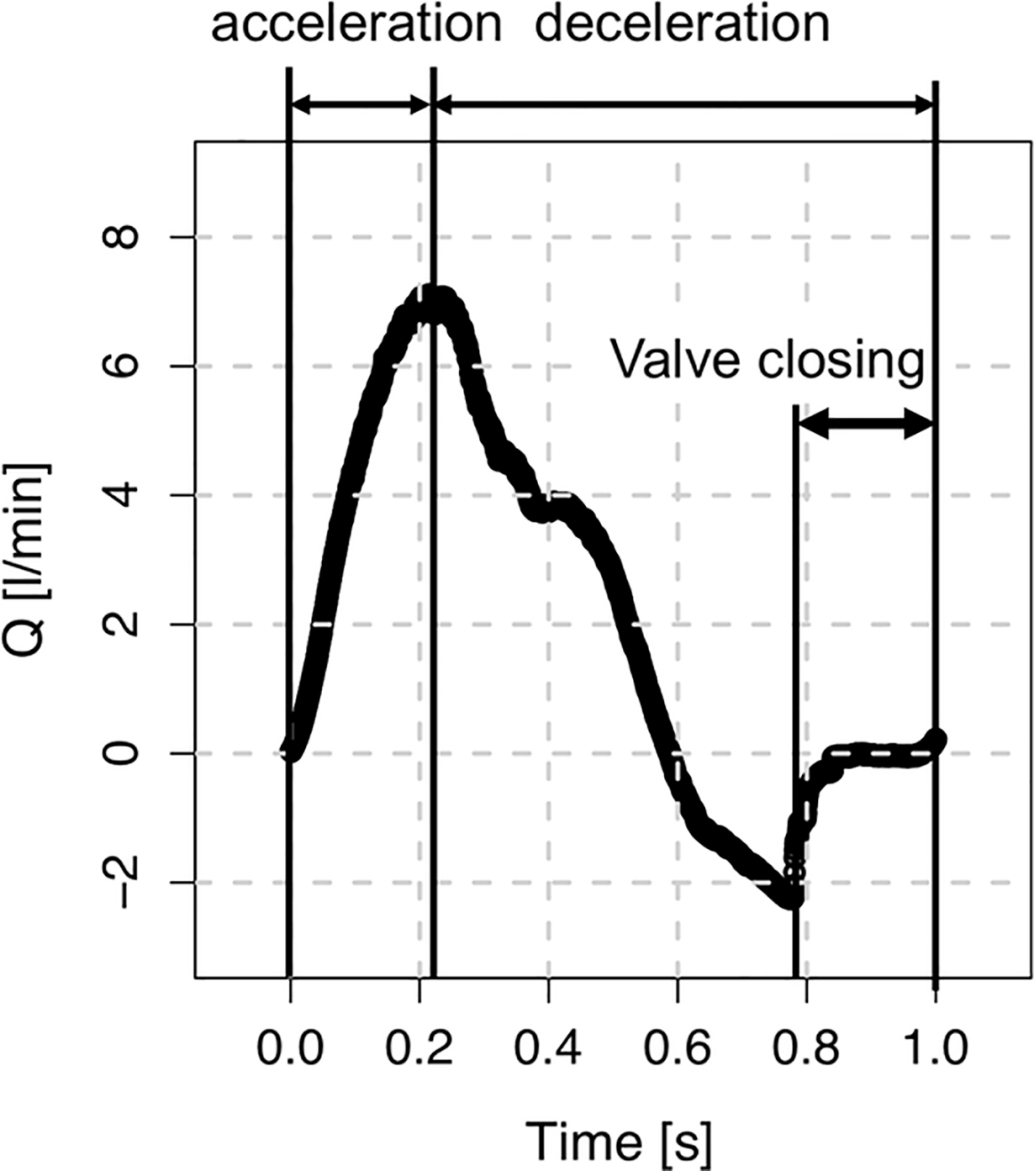
Waveform of the pulsatile inlet flow. Mean and maximum flow rate were 2.0 and 7.1 L/min, which corresponds to Re_mean_ = 779 and Re_max_ = 2707, respectively. Note that the prosthetic valve is open during forward flow, and is closed by a slight portion of the retrograde flow.

The circulating flow by the pulsatile pump induced a periodic pulsatile waveform (Fig 4). Mean and maximum flow rates of the pulsatile flow were 2.0 and 7.1 L/min, which corresponded to *Re*_mean_ = 779 and *Re*_max_ = 2707, respectively, where *Re* is Reynolds number defined as *QD*/*νA* (*Q* is the flow rate, *D* is the diameter of the channel, *ν* is the kinematic viscosity and *A* is the cross-sectional area of the channel).

For pressure measurements, pressure taps of 2 mm in diameter were installed at 70 and 65 mm in the front and behind the pannus location, respectively. The pressure drop between the two points was measured at the constant flow rate of 6 L/min using a U-tube manometer.

### Particle image velocimetry (PIV)

Fig 3B demonstrates the system for measuring PIV velocity fields. To illuminate the measurement plane, we generated a 0.5-mm-thick thin laser sheet using a 1 W continuous diode-pumped solid state laser (Shanghai Dream Lasers Technology Co., Ltd., Shanghai, China). A high-speed camera with 1 × 1 k pixel resolution (Fastcam SA1.1; Photron, San Diego, CA, USA) was used for capturing flow images for velocity field measurements. The high-speed camera consecutively captured approximately 10,917 pairs of flow images in the centre plane of the flow phantom with 2000 frames per second. Instantaneous vector fields obtained from the captured image pairs were divided into 100 different temporal phase angles according to the pulsating cycle. Consequently, 109 instantaneous velocity fields for each cardiac phase angle were ensemble averaged. Detailed step-by-step procedures for obtaining the phase-averaged velocity of vector fields were described previously by Ha and Lee [22, 23].

PIV analysis was performed using PIVlab 1.4[24] built on the MATLAB platform (Mathworks, Natick, MA, USA). A fast Fourier transform-based cross-correlation PIV algorithm was applied to acquired flow images for extracting instantaneous velocity fields. A multigrid interrogation window scheme was adopted using 32 × 32 and 16 × 16 pixels of interrogation windows with 50% overlapping. The distance between two adjacent velocity vectors is six pixels, which corresponds to 0.34 mm. Further details on the principle and measurement uncertainty of the PIV system are described in our previous study [12, 22, 23].

### Transvalvular pressure gradient estimation

To estimate TPG across the prosthetic valve, the simplified Bernoulli equation, which is widely used in the clinical field [25], was used:

$$TPG=4v_{vc}^{2}(mmHg)$$ (1)

where *v*_*vc*_ is defined as the velocity at the vena contracta. In our study, *v*_*vc*_ was calculated as the maximum velocity at the outlet of the prosthetic valve. TPG for each pannus condition were then normalized with that without a pannus to obtain NTPG and clarify the effect of pannus formation on TPG elevation.

### Wall shear stress estimation

WSS is defined as the product of fluid viscosity and shear rate at the vessel wall. To estimate wall locations, we obtained the maximum intensity of each pixel for 10,917 flow image pairs. The obtained image exhibited a bright interior because of tracer particles and a dark exterior outside the conduit. The wall location where the intensity of the image decreased to <5% of the maximum intensity was identified. The shear rate at the wall was estimated using a forward difference scheme and assuming a quadratic velocity profile near the wall. Velocity field data in the near-wall region and the no-slip condition at the wall were used for calculating the velocity gradient (Δu/Δx), which represents the shear rate at the wall. The inherent error in the wall shear rate estimation results from the near wall velocity. As demonstrated in the previous research using the same setup for PIV experiment [5], the error in the centerline velocity was less than ±1%. The error increases to approximately ±5% near the wall. In the nearest wall region, the relative error becomes larger (~±40%) because of the velocity gradient within the interrogation window. Therefore, the WSS has the same level of the accuracy throughout the experiments.

### Principal shear stress

Viscous stress tensor τ_ij_ is defined as follows:

$$\tau_{ij}=\mu \left(\frac{\partial u_{i}}{\partial x_{j}}+\frac{\partial u_{j}}{\partial x_{i}}\right)$$ (2)

where *i* and *j* represent orthogonal directions in the Cartesian coordinates (e.g. *x*, *y* and *z*) and *μ* is the dynamic viscosity. Then, the principal shear stress can be estimated as follows:

$$\tau_{max}=\frac{1}{2}|\sigma_{max}-\sigma_{min}|$$ (3)

where *σ*_*max* and_ *σ*_*min*_ are the principle normal stresses based on the eigenvalues of the viscous stress tensor.

### Viscous energy loss

The viscous dissipation function *Ф*_*v*_ per voxel was calculated from the first order derivatives of the velocity field [26]:

$$\phi_{v}=\frac{1}{2}\sum_{i}\sum_{j}\left[(\frac{\partial u_{i}}{\partial x_{j}}+\frac{\partial u_{j}}{\partial x_{i}})+\frac{2}{3}(\nabla ∙u)\delta_{ij}\right]$$ (4)

where *i* and *j* represent the direction of the Cartesian coordinate and *δ*_*ij*_ is the Kronecker delta. Then, the rate of viscous dissipation ${\dot{E}}_{loss}$ can be estimated by integrating the viscous dissipation function over the total volume:

$${\dot{E}}_{loss}=\mu \sum_{i=1}^{N}\phi_{v}V_{i}$$ (5)

where *V*_*i*_ is the volume of each voxel and *N* is the total number of voxels within the volume. Because PIV measurement used in our study provided only a two-dimensional velocity field, Ф_v_ in Eq (4) was obtained assuming that the flow is two-dimensional and calculation of ${\dot{E}}_{loss}$ was confined to the centre plane of the aortic model. The depth-directional size of the voxel was assumed to be the same with the *x* and *y* pixel-spacing size.

### Analysis of the opening angle of the prosthetic valve

The opening angle of the prosthetic valve under each experimental condition was observed from the cross-sectional view using a high-speed camera at 125 frames per second. The leaflet areas in all cross-sectional images were manually masked for identifying the projection length of the leaflet. Thereafter, the opening angle of the prosthetic valve was obtained using the cosine rule (θ = cos^−1^[L/L_0_]), where L is the projection length at the opening state and L_0_ is the length of the leaflet (S1 Fig). In our study, maximum opening angles of both leaflets during five cardiac cycles were used to obtain the mean ± standard error. For statistical analysis of the opening angle, the statistical difference of the opening angle at each W/D was compared with that at W/D = 0 using Student’s *t*-test. It was performed using SPSS statistical software (SPSS Inc., Chicago, IL, USA). Significance level was set at p < 0.05.

## Results

### Opening angle of the prosthetic valve and extent of the pannus

**Fig 5:**
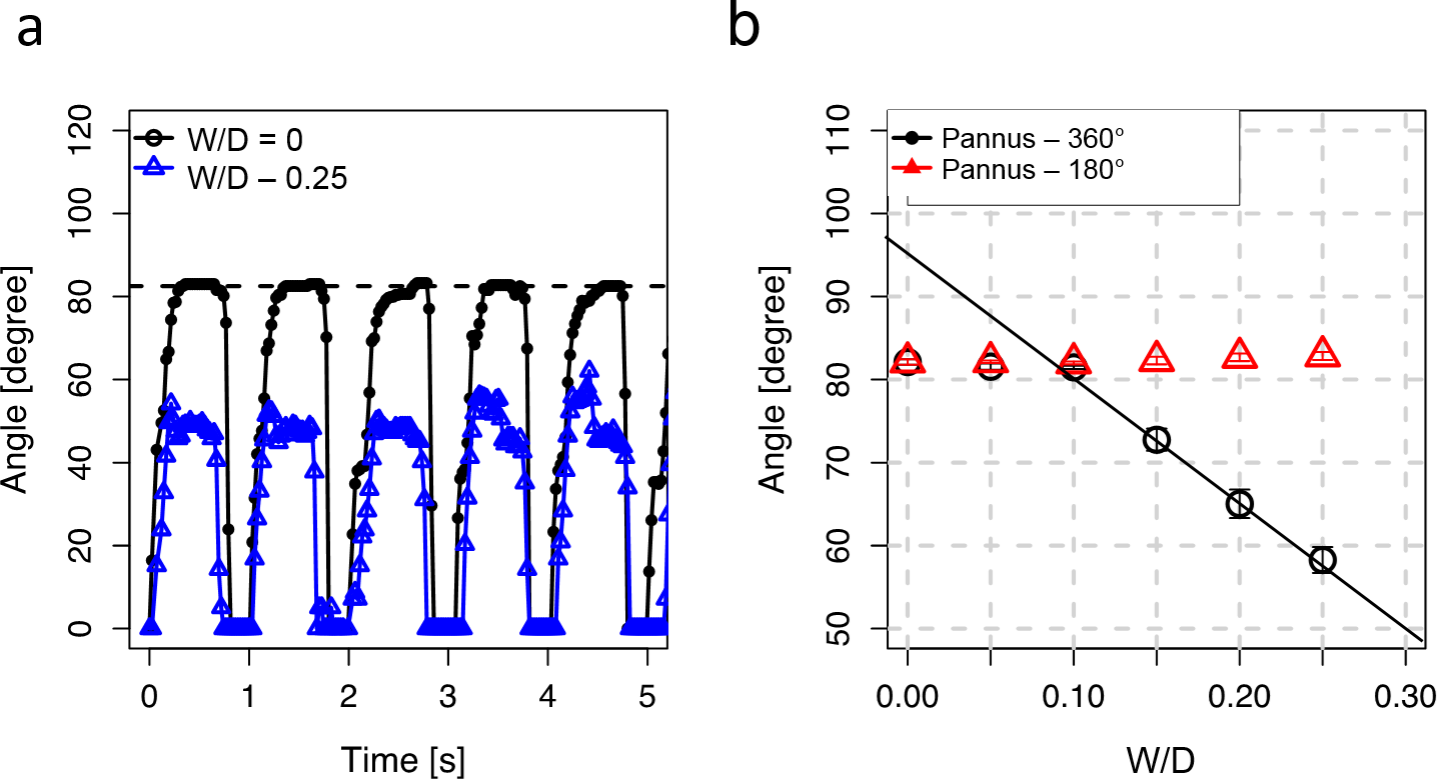
Relationship between pannus formation and the opening angle of the prosthetic valve. (a) Estimation of maximum opening angle of the prosthetic valve at W/D = 0. Maximum opening angles of both leaflets during five cardiac cycles were used to obtained mean ± SE. (b) Effect of W/D on the maximum opening angle. Insets indicate opening of the mechanical valve at W/D = 0 and 0.25. All opening images of the prosthetic valve can be seen in S2 Fig.

The prosthetic valve showed periodic opening and closing of the leaflets under pulsatile flow conditions (Fig 5A). When the ratio of pannus width to valve inner diameter (W/D) was zero, the maximum opening angle of the prosthetic valve was approximately 82°, which was close to the normal opening angle of the St. Jude prosthetic valve around 85°. Conversely, pannus formation with an involvement angle of 360° (Pannus-360°) and W/D = 0.25 had a significantly reduced opening angle of 58.3 ± 1.6° (Fig 5A). Although pannus formation with W/D < 0.1 did not induce significant changes in the opening angle compared with W/D = 0 (p > 0.05; Fig 5B), Pannus-360° markedly decreased the maximum opening angle of the prosthetic valve at W/D > 0.1 (p < 0.01; 72.8 ± 1.3° at W/D = 0.15, 65.0 ± 1.7° at W/D = 0.2, and 58.3 ± 1.6° at W/D = 0.25; S2 Fig). Linear regression of the opening angle of the prosthetic valve with Pannus-360° (W/D ≥ 0.10) yielded the following equation: opening angle = 94.65 − 154.67 × W/D. Conversely, pannus formation with an involvement angle of 180° (Pannus-180°) did not induce a significant decrease in the maximum opening angle of the prosthetic valve at 0 ≤ W/D ≤ 0.25.

### Transvalvular velocity and pressure gradient

**Fig 6:**
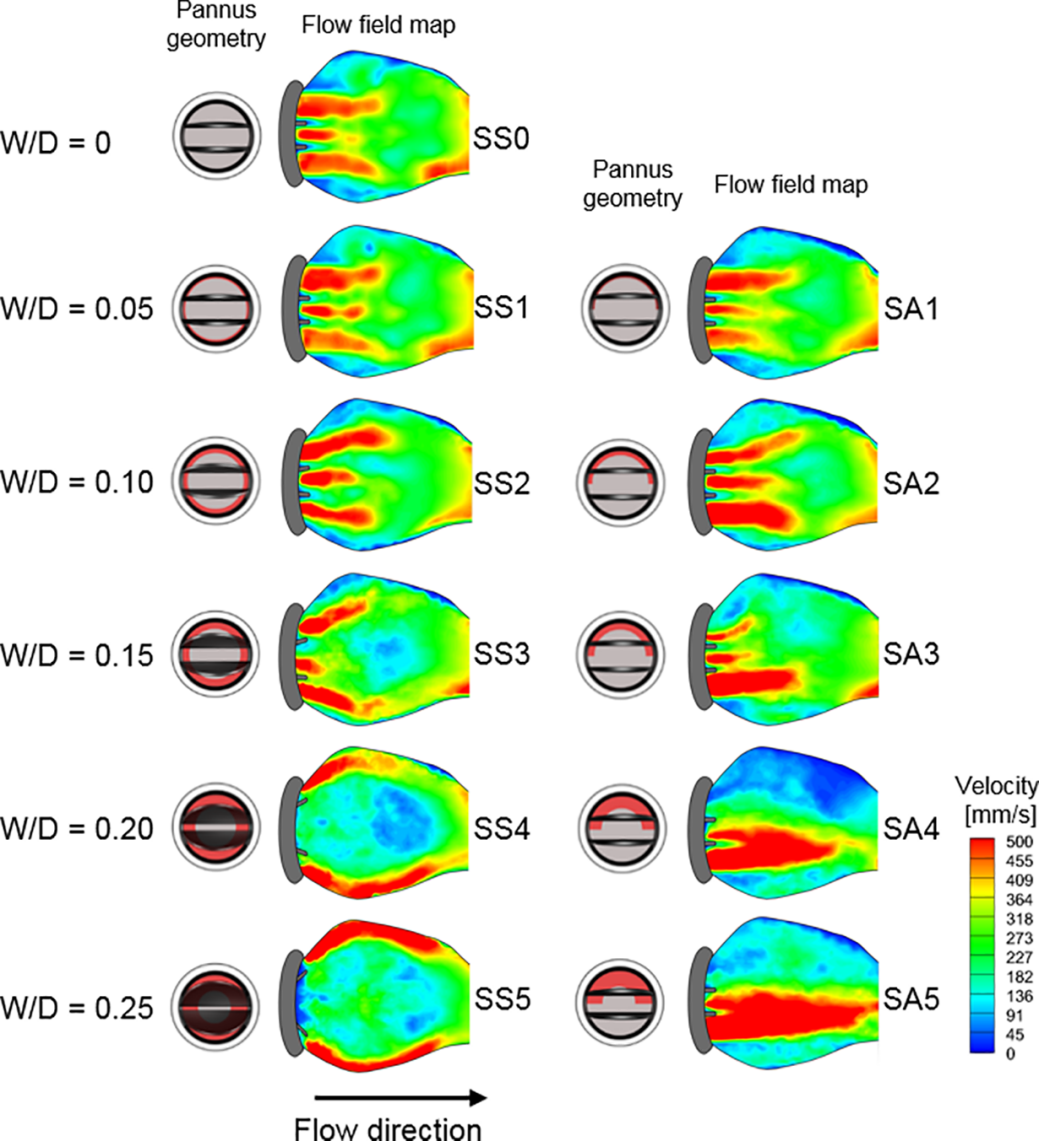
Effects of pannus involvement (Pannus 360° vs. Pannus 180°) on the field behind the prosthetic valve at peak systole. Note that only results with the straight inlet case are shown and the results with 25% inlet obstruction are included in S3 Fig.

The velocity field at the postprosthetic valve significantly varied according to the extent of pannus formation. Without pannus formation (W/D = 0), the flow field at the outlet of the prosthetic valve had three separate jet flows coming from two sides and one central orifice (Fig 6). Because W/D of Pannus-360° increased (left column of Fig 6), two jet flows from the side orifices were directed towards the aortic sinus wall and the central jet flow was obstructed because of the reduced opening angle of the prosthetic valve. Conversely, Pannus-180° generated a straight, forward flow regardless of W/D and the one-sided flow coming through the pannus was dramatically reduced because the eccentric pannus (Pannus-180°) obstructed the corresponding region (right column of Fig 6). Changes in the inlet velocity profile with a 25% inlet obstruction did not induce significant changes in hemodynamic features, and the velocity fields were mostly dependent on the pannus geometry (S3 Fig).

**Fig 7:**
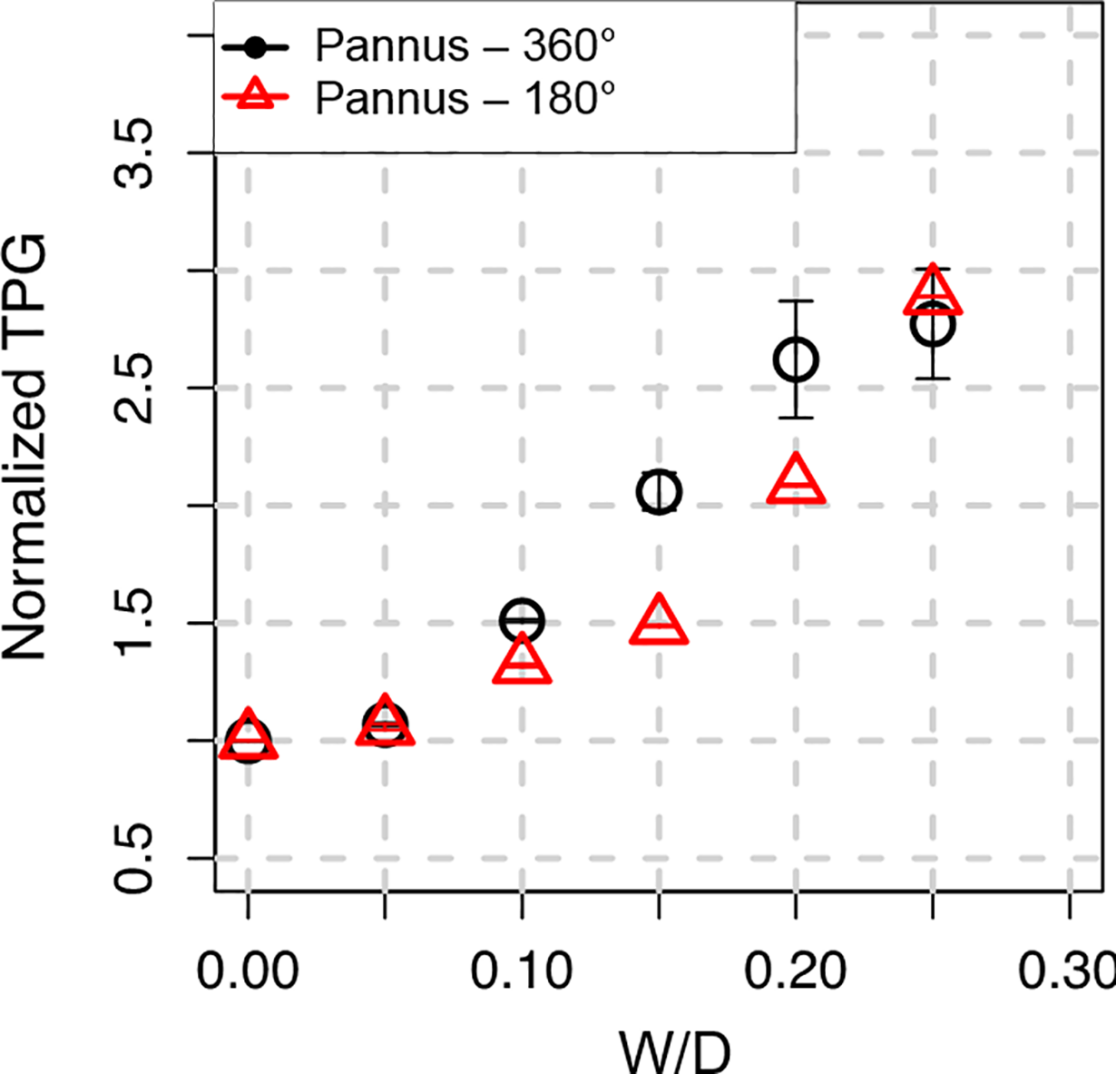
Relationship between W/D and NTPG. NTPG is obtained by dividing TPG by TPG obtained at W/D = 0. TPG at W/D = 0 was 2.1 mmHg.

Subvalvular pannus formation resulted in substantial changes in the transvalvular peak velocity and corresponding transvalvular pressure gradient (TPG) (Figs 6 and 7). The increase in W/D resulted in an parabolic increase in TPG for Pannus-360° and Pannus-180°, mostly due to an increase in the maximum velocity at the outlet of the prosthetic valve, as shown in Figs 3 and 4. Regardless of pannus involvement angle, TPG at W/D = 0.25 was more than approximately 2.5 times higher than that without pannus formation (W/D = 0). The quadratic relationship between TPG and W/D was obtained for normalized TPG (NTPG), which was normalized by TPG at W/D = 0; NTPG = 0.89 + 6.44 × W/D + 6.37 × W/D^2^ for Pannus 360° (p < 0.001), NTPG = 1.04 − 1.80 × W/D + 36.20 × W/D^2^ for Pannus 180° (p < 0.001), and NTPG = 0.96 + 2.32 × W/D + 21.29 × W/D^2^ for all pannus conditions (p < 0.001). Results showed that TPG of Pannus 180° (0 ≤ W/D ≤ 0.25) had a quadratic correlation with W/D, although the opening angle of the prosthetic valve with Pannus-180° was not correlated with W/D (Figs 5, 6 and 7). Changes in the inlet velocity profile with a 25% inlet obstruction did not induce significant changes in NTPG and they were mostly dependent on W/D of the pannus formation (S4 Fig). The hemodynamic parameters including the velocity, NTPG and WSS were also described in Table 2.

**Table 2:** Summary of hemodynamic parameters.

Case	W/D	Velocity [mm/s]	Normalized TPG	WSS [N/m^2^, upper wall]	WSS [N/m^2^, lower wall]
SS0	0.00	559.9±1.8	1.00±0.00	-0.39±0.39	-0.43±0.16
SS1	0.05	578.7±4.6	1.07±0.00	-0.69±0.38	-0.74±0.26
SS2	0.10	668.2±5.4	1.52±0.00	-0.67±0.30	-0.79±0.40
SS3	0.15	803.4±21.7	2.07±0.00	0.59±0.58	-0.69±0.45
SS4	0.20	906.5±43.3	2.63±0.01	1.63±0.67	1.15±0.73
SS5	0.25	932.2±43.1	2.78±0.01	1.84±0.76	2.04±1.08
SA1	0.05	576.1±6.4	1.06±0.00	-0.56±0.40	-0.51±0.30
SA2	0.10	643.4±4.9	1.32±0.00	-0.69±0.34	-0.93±0.28
SA3	0.15	682.9±4.7	1.49±0.00	-0.65±0.21	-0.62±0.29
SA4	0.20	808.7±5.2	2.09±0.00	-0.62±0.25	-0.34±0.20
SA5	0.25	951.7±4.5	2.90±0.00	-0.64±0.38	-0.24±0.14

### Wall shear stress, principal shear stress and viscous energy loss

**Fig 8:**
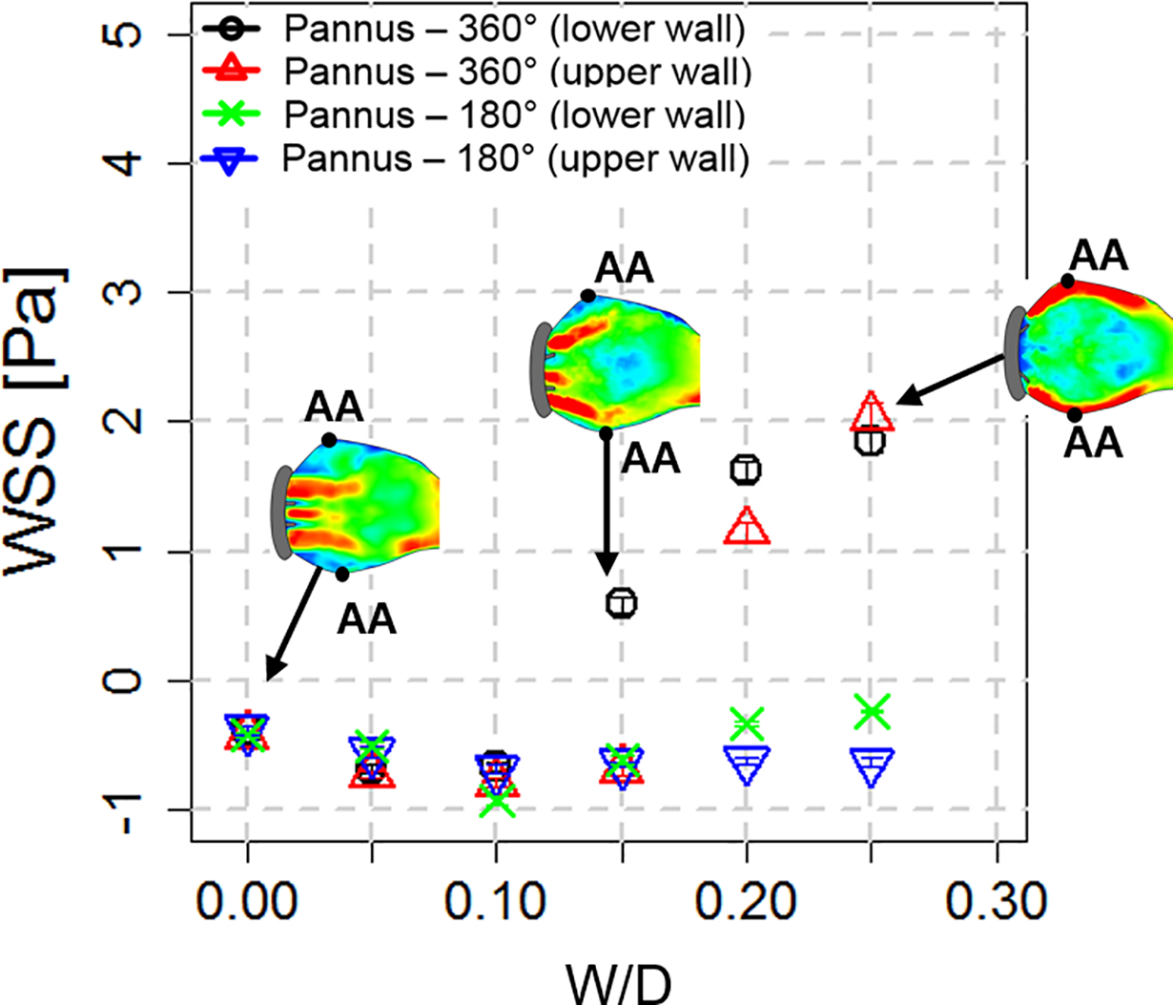
Relationship between W/D and WSS at the aortic sinus. Note that Pannus 360° with W/D > 0.15 tends to produce positive WSS at the aortic sinus, whereas Pannus-180° produces negative WSS. Each data value indicates mean ± SE of WSS measured at the aortic sinus (AA).

The sinus wall without pannus formation (W/D = 0) had a low negative wall shear stress (WSS) (approximately 0.4 N/m^2^) at peak systole because retrograde recirculation flow developed near the sinus wall. Compared with W/D = 0, Pannus-360° with W/D > 0.1 dramatically increased WSS up to approximately 2.0 N/m^2^ because it induced the high-velocity forward jet flow towards the sinus wall. Conversely, Pannus-180° consistently induced negative WSS regardless of W/D (Fig 8).

**Fig 9:**
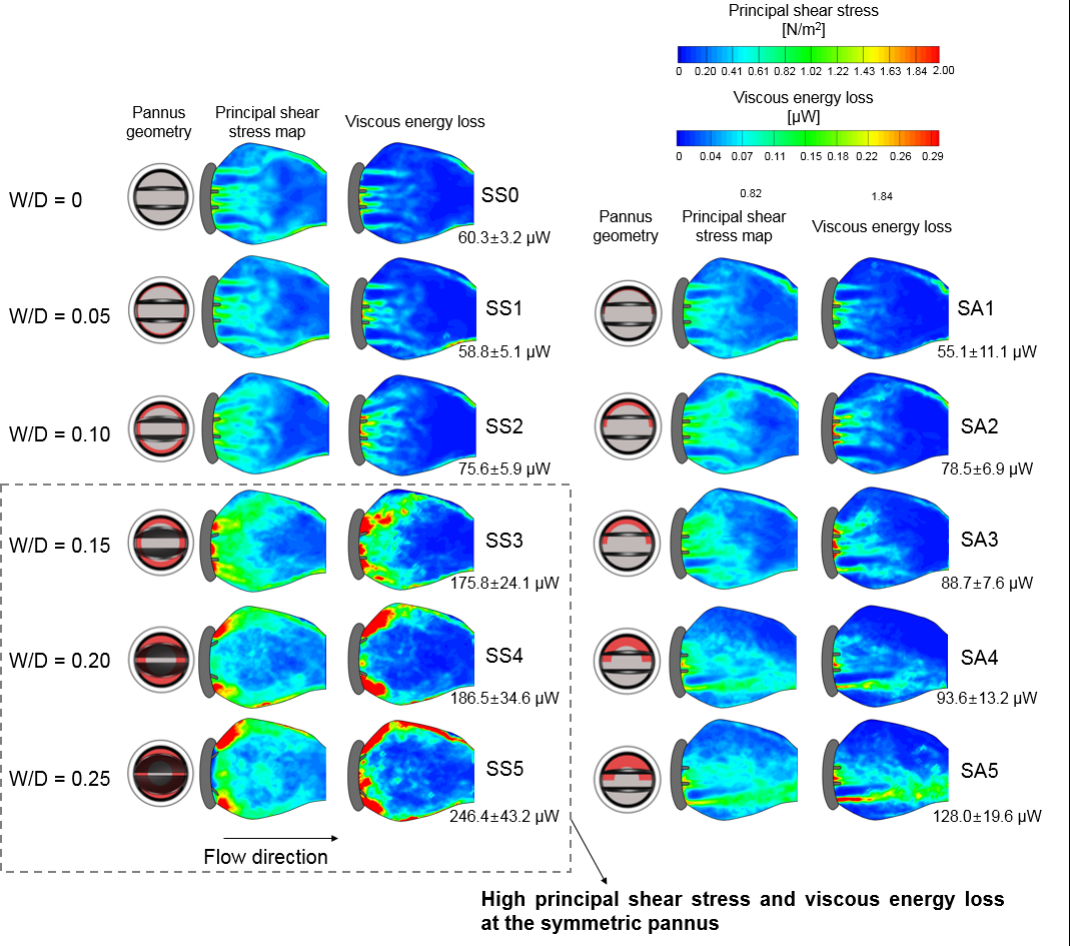
Effects of pannus formation on principal shear stress and corresponding viscous energy loss behind the prosthetic heart valve. The numeric value of total viscous energy loss within the aortic sinus was shown right below the viscous energy loss mapping. Note that only results with the straight inlet case are shown and results with 25% inlet obstruction are included in S5 and S6 Figs.

Fluid dynamic changes due to pannus formation directly influenced principal shear stress and viscous energy loss at the aortic sinus (Fig 9). The maximum principal shear stress and viscous energy loss at W/D = 0 was approximately 1.5 N/m^2^ and 0.3 μW (per voxel), respectively; however, an increase in W/D (W/D = 0.25) elevated the maximum principal shear stress and viscous energy loss to 3.8 N/m^2^ and 3.1 μW (per voxel), respectively. Although Pannus-360° and Pannus-180° resulted in similar maximum viscous energy loss at the same W/D, the extent of viscous energy loss differed by the pannus type and its effect on the flow structure. Consequently, at W/D > 0.15, the total viscous energy loss within the aortic sinus at Pannus-360° was approximately two times higher than that at Pannus 180° (Fig 9, S5 and S6 Figs).

**Fig 10:**
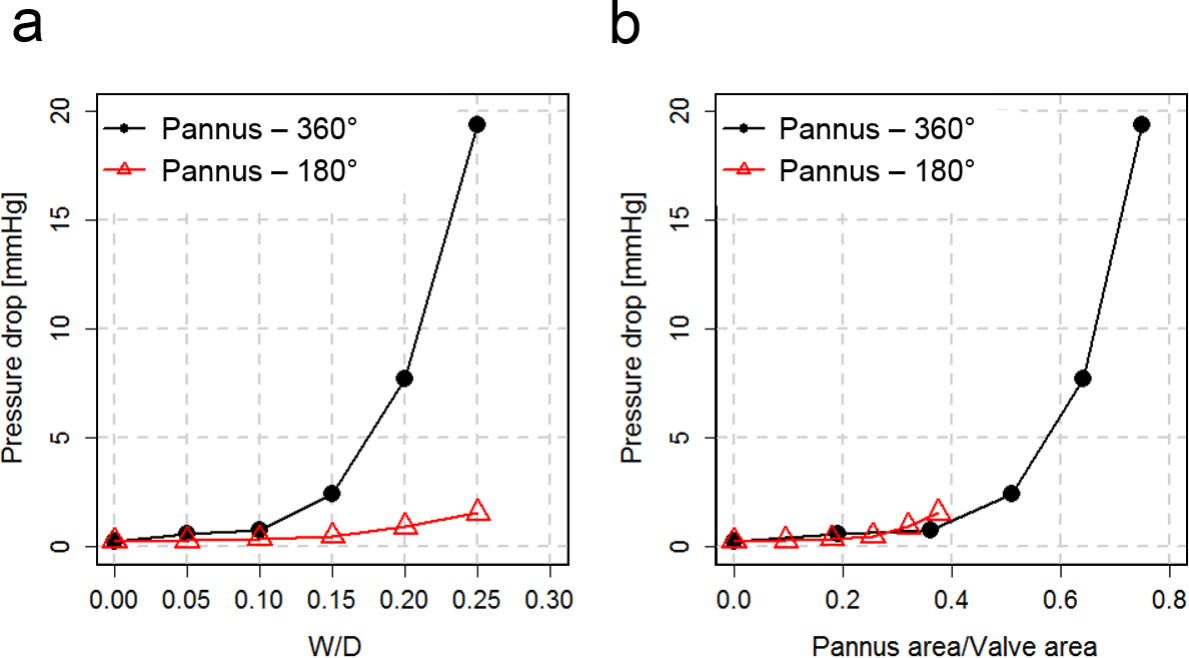
Relationship between pannus formation and pressure drop across the prosthetic valve. (a) The relationship between the pressure drop and pannus width. (b) The relationship between the pressure drop and pannus area. The flow rate was fixed to constant of 6 L/min. Note that pressure ports for the pressure measurement were located at 70 and 65 mm in front and behind the pannus, respectively.

The discrepancy between Pannus-360° and Pannus-180° was also observed by direct pressure measurement (Fig 10). The pressure drop across the prosthetic valve at a constant flow rate of 6 L/min with Pannus-360° dramatically increased as W/D increased, reaching up to 19.4 mmHg at W/D = 0.25. Conversely, Pannus-180° induced only a minor increase in the pressure drop (1.6 mmHg at W/D = 0.25). On the other hand, the pressure drop across the prosthetic valve was mostly affected by the pannus area.

## Discussion

We investigated the effect of pannus formation on prosthetic valve mechanical function and hemodynamics in the aortic sinus and their clinical implications using an in vitro flow phantom demonstration with PIV measurement. Major findings of this study were as follows: Subvalvular pannus formation resulted in substantial changes in the transvalvular peak velocity, TPG and opening angle of the prosthetic valve. The velocity flow field at the aortic sinus and corresponding hemodynamic indices, including WSS, principal shear stress and viscous energy loss distributions, were largely influenced by the pannus involvement angle (Pannus-360° vs. Pannus-180°). The maximum flow velocity and corresponding TPG were affected by pannus width and were not affected by the pannus involvement angle. Consequently, a substantial discrepancy between the velocity-based TPG estimation and direct pressure measurements was observed for prosthetic valve flow with the pannus.

Our study showed that an increase in Pannus 180° (0 ≤ W/D ≤ 0.25) resulted in an elevation in TPG, but the prosthetic valve could still have normal valve motion. Pannus-180° increased maximum flow velocity at the outlet of the valve by biasing flow towards the unobstructed orifice, which consequently resulted in an increase in TPG. Considering that previous studies reported that approximately 40% patients with pannus had normal valve motion, the elevation of TPG by pannus formation does not always imply prosthetic valve dysfunction [27, 28]. Our study confirmed that pannus formation can influence TPG across the valve regardless of the valve mechanical function.

**Fig 11:**
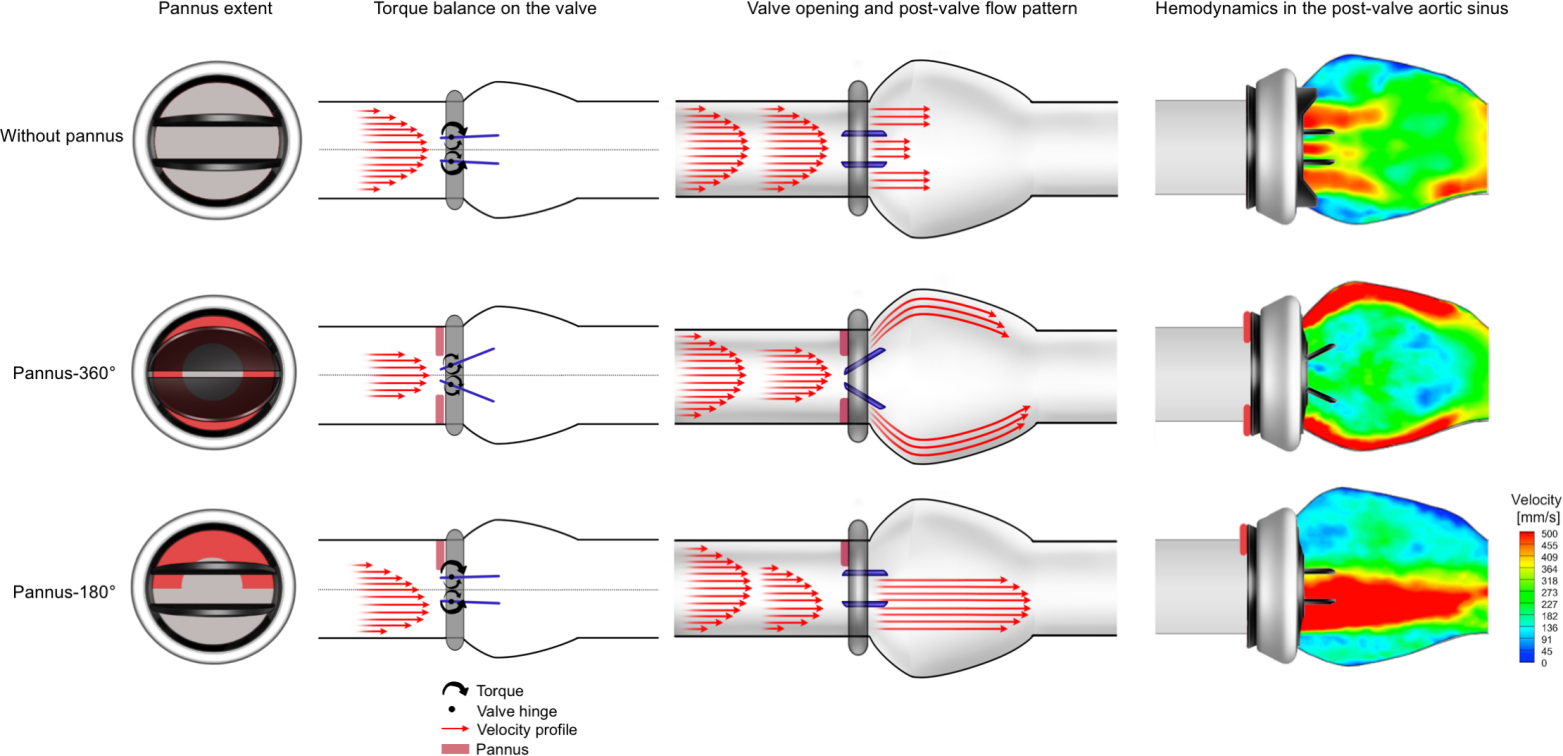
Graphical summary of pannus formation and its effects on valvular dysfunction and hemodynamics in the post-valve aortic sinus.

Opening angles of the prosthetic valve were different depending on the pannus involvement angle (Pannus-180° vs. Pannus-360°). It has been probably caused by the different torque balance exerted on the valve leaflets depending on the pannus type (Fig 11). The circumferential pannus (Pannus-360°) induces a high-velocity gradient jet flow towards the valve leaflets. The central high-velocity component of the flow exerts torque towards the closing direction opposite the valve opening. Consequently, the balance of the torque establishes at a certain opening angle so that the leaflets do not open completely. As W/D increases, the central jet becomes stronger, which exerts a stronger closing directional torque, reducing the maximum opening angle of the prosthetic valve. Conversely, the Pannus-180° generates an off-axis velocity profile with a relatively lower velocity gradient because of a larger effective orifice area. Therefore, the closing directional torque generated by the Pannus-180° was much lower than the opening directional torque. Therefore, the valve leaflets are completely opened. To prove the hypothesis, further in-depth studies on the fluid dynamics near valve leaflets with pannus formation are needed. However, as conventional optical imaging cannot visualize the internal flow within an opaque prosthetic valve, alternative imaging techniques such as fluoroscopic imaging or X-ray PIV will be required [29].

This study found substantial discrepancy between velocity-based TPG estimation and direct pressure measurements for prosthetic valve flow with the pannus. As shown in Figs 6 and 7, the peak velocity of the flow and corresponding TPG were more dependent on W/D than on pannus involvement angle. However, direct pressure measurement showed that the relationship between the pressure drop and W/D largely varied depending on the pannus involvement angle (Fig 10). The pressure drop across the prosthetic valve was rather affected by the pannus area. This discrepancy was caused by the complex flow structures generated by each pannus. Although the conventional simplified Bernoulli equation for TPG estimation was optimized for the single directional jet flow from the orifice, the prosthetic valve with pannus formation generated multidirectional complex flow. Pannus-360° generated two directional jet flows from two partially closed leaflets, whereas Pannus-180° generated a single, straight jet blood flow. These complex flow structures that were generated by each pannus influenced viscous energy loss at the aortic sinus, which is one of the important parameters influencing the energy loss in fluid dynamics (Fig 9).

Our results demonstrated the need for additional fluid dynamic information (e.g. principal shear stress and viscous energy loss) for diagnosing the risk of prosthetic valve with pannus formation more accurately. Recently, four-dimensional (4D) phase-contrast magnetic resonance imaging (PC-MRI) has been introduced as a useful method for quantifying temporal variations in three-dimensional velocity flow fields [30]. Because 4D PC-MRI can measure fluid dynamic parameters, including WSS, pressure difference, turbulent kinetic energy and viscous energy loss, in patients with good accuracy [26, 30], it would be useful for determining the risk of prosthetic valve with pannus formation when used as supplementary information with conventional TPG estimation.

It is also noteworthy that partial closing of the prosthetic valve significantly increased WSS at the aortic sinus wall (Fig 8). Spatial and temporal alterations in WSS on the endothelium affect arterial remodelling [31]. In particular, recent studies revealed that an increase in WSS in the ascending aorta by the bicuspid aortic valve is associated with aortic dilatation [32, 33]. Because the aortic sinus in a normal physiological condition is usually exposed to retrograde WSS [34, 35], abnormal changes in WSS from low negative to high forward WSS may contribute to abnormal sinus remodelling, such as dilation. However, clinical evidence regarding pannus formation and sinus malformation is lacking and in-depth studies would be required in the future.

Due to the simplification of the in-vitro experiments, the current results may not exactly replicate the in-vivo conditions, but they still provide a first approximation of the effects of pannus formation, which provide clinically meaningful relationships between the pannus formation and hemodynamic changes. Despite previous CT demonstration of pannus formation, the relationship between prosthetic valve dysfunction and the extent of pannus involvement remains unclear. Until lately, it was not clear if the pannus size can be a clinical index determining hemodynamic dysfunction [20]. The present study found that the pannus formation resulted in substantial changes in the transvalvular peak velocity, TPG and opening angle of the prosthetic valve. The size and extent of the pannus were found to be important parameters. This relationship was also confirmed in the latest publication with in-vivo patients [20]. Therefore, we believe that our in-vitro experiments capture clinically relevant results.

While the present study found that the pannus formation resulted in substantial changes in the transvalvular peak velocity, TPG and opening angle of the prosthetic valve, the quantification of the pannus formation for clinical practices needs further consideration due to the geometric complexity of the pannus formation in the patients. We suggest the researchers need to calculate both maximum and average value of each pannus parameter along the circumferential direction. Referring to the previous study [20], both maximum and average values of pannus width, pannus width/valve diameter and pannus area would be helpful to quantify the pannus formation for the in-vivo patients.

The limitation of the present study was that the pulsatile flow used in this study was not completely same with the heart flow in human subjects. Therefore, the maximum and mean flow rates used in the present study were slightly smaller than physiological values. However, the overall trend in pressure and TPG vs. W/D were not significantly affected by the flow conditions. Although preliminary studies have found that the opening angles of the prosthetic valve were more dependent on W/D rather than the flow conditions, more physiological conditions should be investigated in the further studies. In addition, pressure drop was measured under a constant flow condition, whereas TPG was calculated under the pulsatile flow condition. Therefore, direct comparison of the pressure and TPG is not applicable. However, overall trends in the pressure and TPG versus W/D were not significantly affected by flow conditions because preliminary studies found that prosthetic valve opening angles were dependent on W/D than on flow conditions. The aortic geometry used in this study was straight, rigid and symmetric. Since the native aorta is arched, compliant, and asymmetric, these differences may affect the flow field in the aorta thus should be considered in the future study. Lastly, due to the opacity of the prosthetic valve, the current study could not analyze the fluid dynamics in the hinge region of the prosthetic valve. As we consider that the torque or force balances on the hinge region highly influences the function of the prosthetic valve, this aspect should be investigated in the future study

## Supporting information

S1 Fig Estimation of the opening angle of the prosthetic valve from the cross-sectional view. Because the cross-sectional projection image provides L_0_ and L, the corresponding angle of the prosthetic valve can be obtained from θ = cos^−1^(L/L_0_).
(TIF)

S2 Fig Opening images of the prosthetic valve with various pannus formations at peak systole. (TIF)

S3 Fig Effects of pannus involvement (Pannus-360° vs. Pannus-180°) on the field behind the mechanical valve. Note that 25% inlet obstruction was used for the inlet condition.
(TIF)

S4 Fig Relationship between W/D and NTPG. The relationship between W/D and NTPG shows that the increase in TPG is not sensitive to inlet flow changes because of 25% inlet obstruction. NTPG = 0.8651 + 7.8299 × W/D + 3.0779 × W/D^2^, Pannus 360°, 25% inlet obstruction; NTPG = 0.8389 − 0.4343 × W/D + 38.0332 × W/D^2^, Pannus 180°, 25% inlet obstruction (upper); and NTPG = 1.032 − 8.817 × W/D + 74.877 × W/D^2^, Pannus 180°, 25% inlet obstruction (lower).
(TIF)

S5 Fig Effects of pannus involvement (Pannus-360° vs. Pannus-180°) on the principal stress field behind the prosthetic heart valve. Note that 25% inlet obstruction was used for the inlet condition.
(TIF)

S6 Fig Effects of pannus involvement (Pannus-360° vs. Pannus-180°) on the viscous energy loss behind the prosthetic heart valve. (TIF)
