# Supplementary figures and images for: Effect of pannus formation on the prosthetic heart valve: In vitro demonstration using particle image velocimetry
Source: PLoS One. 2018 Jun 28;13(6):e0199792. doi: 10.1371/journal.pone.0199792 (PMC6023143; doi:10.1371/journal.pone.0199792)

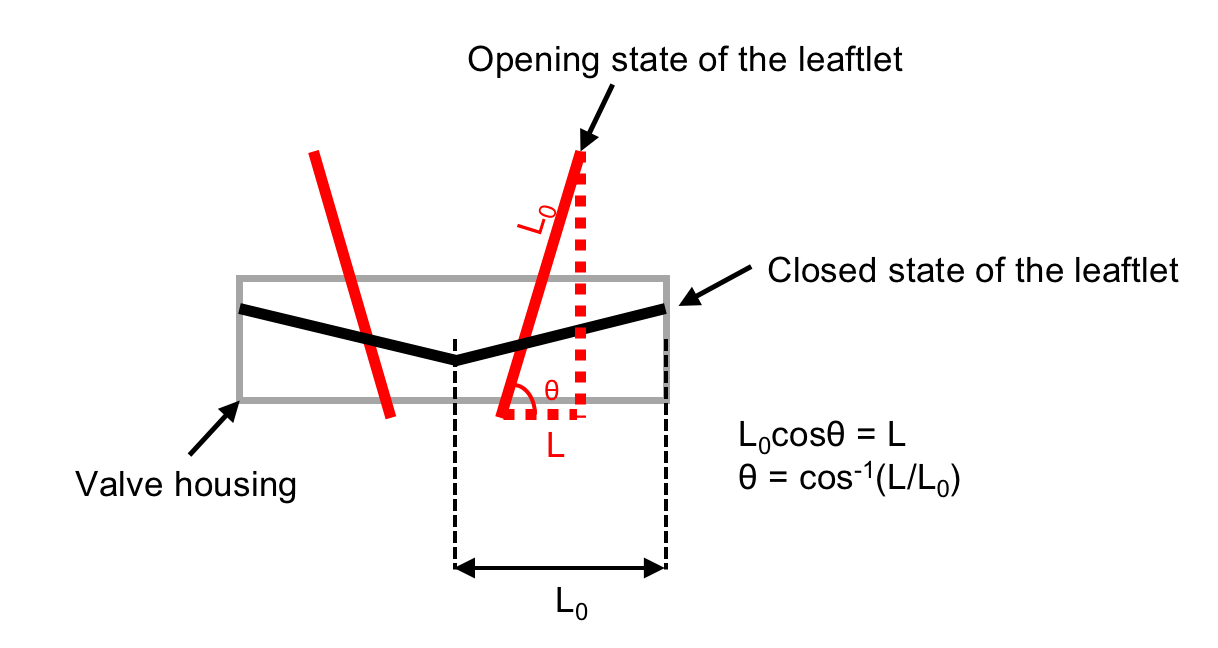

Supplement: S1 Fig — Because the cross-sectional projection image provides L0 and L, the corresponding angle of the prosthetic valve can be obtained from θ = cos−1(L/L0). (TIF) [file pone.0199792.s001.tif]

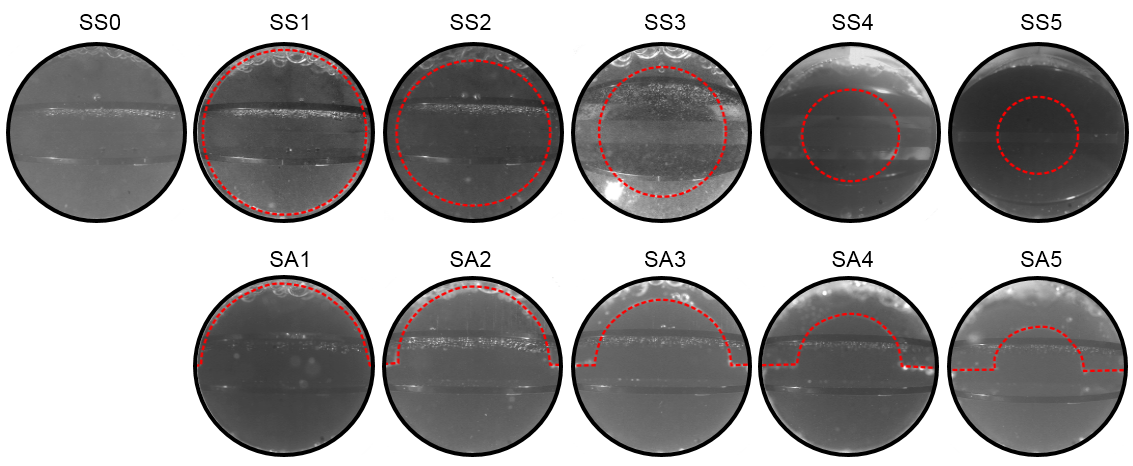

Supplement: S2 Fig — (TIF) [file pone.0199792.s002.tif]

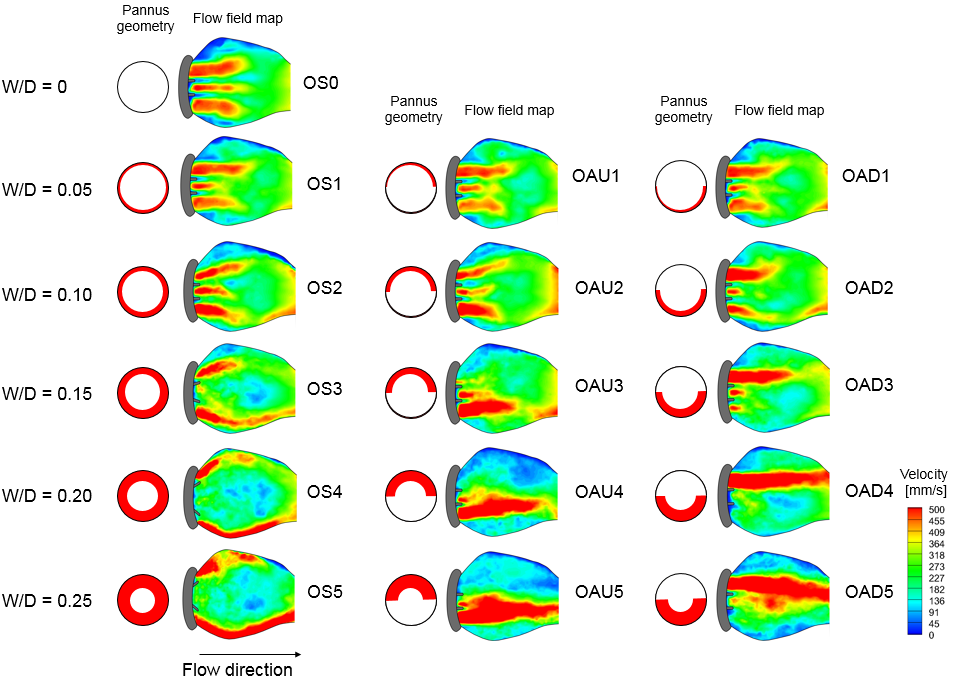

Supplement: S3 Fig — Note that 25% inlet obstruction was used for the inlet condition. (TIF) [file pone.0199792.s003.tif]

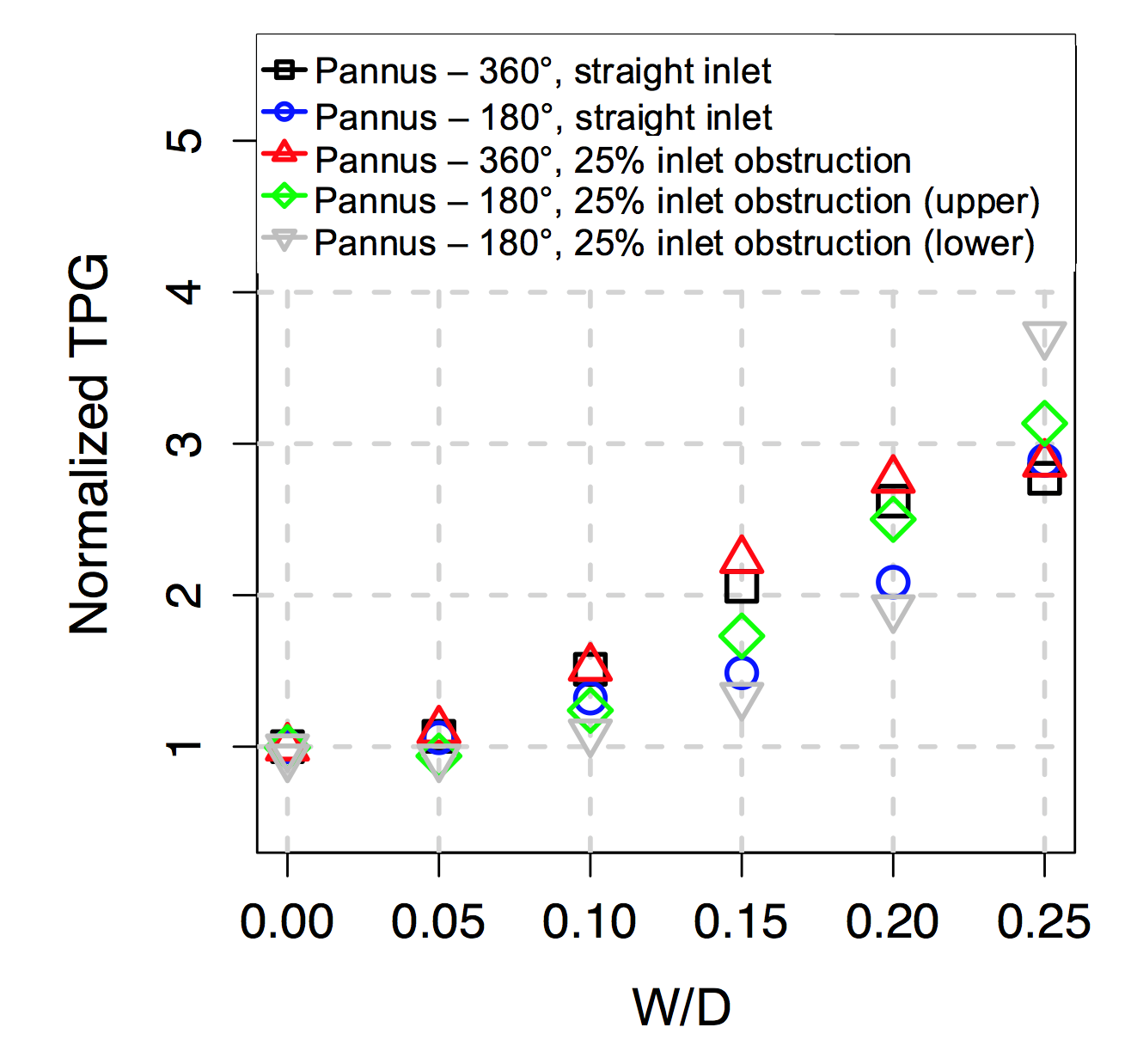

Supplement: S4 Fig — The relationship between W/D and NTPG shows that the increase in TPG is not sensitive to inlet flow changes because of 25% inlet obstruction. NTPG = 0.8651 + 7.8299 × W/D + 3.0779 × W/D2, Pannus 360°, 25% inlet obstruction; NTPG = 0.8389 − 0.4343 × W/D + 38.0332 × W/D2, Pannus 180°, 25% inlet obstruction (upper); and NTPG = 1.032 − 8.817 × W/D + 74.877 × W/D2, Pannus 180°, 25% inlet obstruction (lower). (TIF) [file pone.0199792.s004.tif]

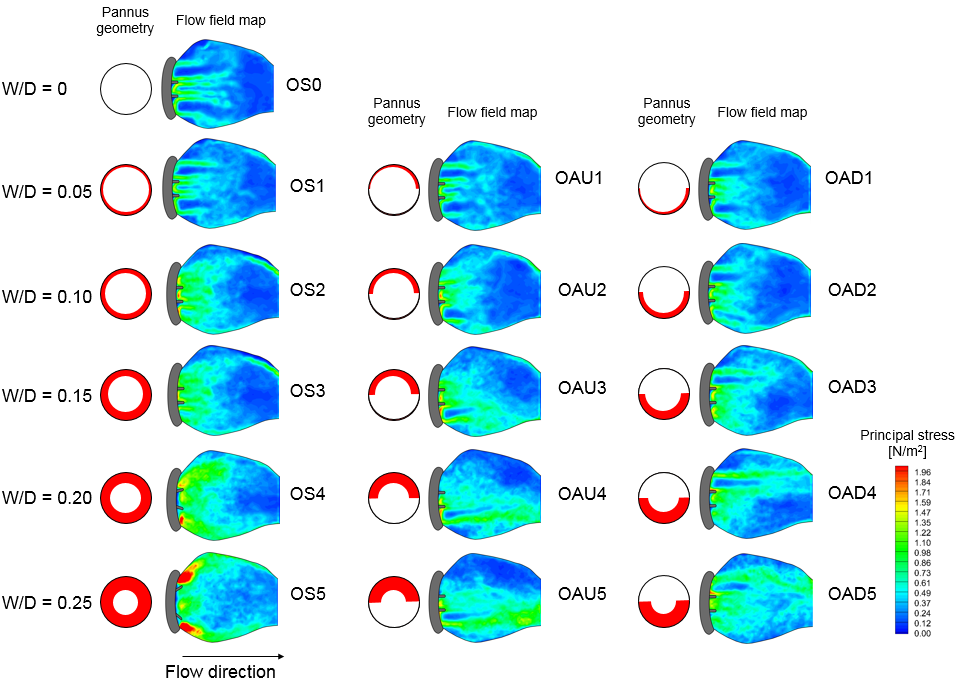

Supplement: S5 Fig — Note that 25% inlet obstruction was used for the inlet condition. (TIF) [file pone.0199792.s005.tif]

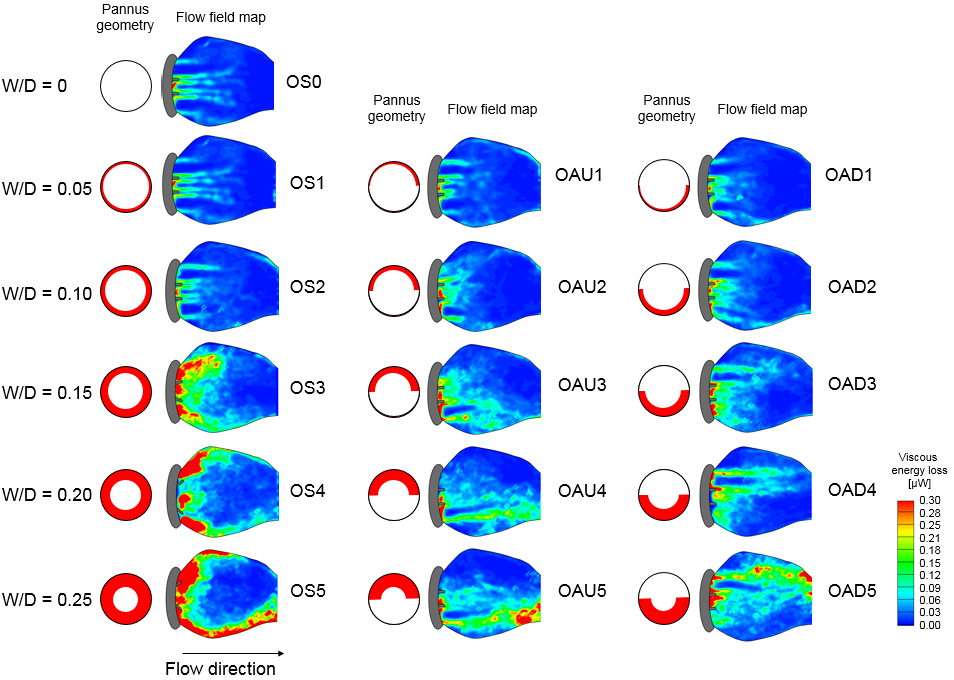

Supplement: S6 Fig — (TIF) [file pone.0199792.s006.tif]
